# A Fluctuating State in the Framework Compounds (Ba,Sr)Al_2_O_4_

**DOI:** 10.1038/srep19154

**Published:** 2016-01-13

**Authors:** Yui Ishii, Hirofumi Tsukasaki, Eri Tanaka, Shigeo Mori

**Affiliations:** 1 Department of Materials Science, Osaka Prefecture University, Sakai, Osaka 599-8531, Japan

## Abstract

The structural fluctuation in hexagonal Ba_1−*x*_Sr_*x*_Al_2_O_4_ with a corner-sharing AlO_4_ tetrahedral network was characterized at various temperatures using transmission electron microscopy experiments. For *x* ≤ 0.05, soft modes of ***q*** ~ (1/2, 1/2, 0) and equivalent wave vectors condense at a transition temperature (*T*_C_) and form a superstructure with a cell volume of 2*a* × 2*b* × *c*. However, *T*_C_ is largely suppressed by Sr-substitution, and disappears for *x* ≥ 0.1. Furthermore, the ***q*** ~ (1/2, 1/2, 0) soft mode deviates from the commensurate value as temperature decreases and survives in nanoscaled regions below ~200 K. These results strongly suggest the presence of a new quantum criticality induced by the soft mode. Two distinct soft modes were observed as honeycomb-type diffuse scatterings in the high-temperature region up to 800 K. This intrinsic structural instability is a unique characteristic of the framework compound and is responsible for this unusually fluctuating state.

Novel phases near the ordered states of spins, charges, or orbitals of electrons have long been fascinating subjects in condensed matter physics. Notable examples are superconductivity near an antiferromagnetic order, a charge density wave (CDW), a spin density wave (SDW), and an orbital order, which are typically found in cuprates, transition metal dichalcogenides^1^, iron arsenides^2^, and ruthenates^3^. The accumulated knowledge from these studies has provided us with the simple and universal description that an extraordinary state often emerges from the fluctuation in an ordered state of the spins, charges, or orbitals. Just as an electron, a phonon is a fundamental quantum in a crystal. Despite a large number of experimental and theoretical studies that focus on fluctuations in these three aspects of electrons, there are few studies on the new phenomenon in which the “fluctuation in phonons” plays a key role.

In framework compounds containing linked polyhedra, for example, several modifications of silicates^4^,^5^, nepheline^6^, and ZrW_2_O_8_^7^, the existence of correlated motions of polyhedra within the network structure has been reported. These correlated motions are called “rigid unit modes” (RUMs)^8^,^9^,^10^. These RUMs sometimes act as a soft mode that induces a structural phase transition, for example, the structural phase transitions in quartz^4^, tridymite^5^, and nepheline^6^. For the framework compound BaAl_2_O_4_, it has also been argued that a RUM is the dominant structural instability^11^.

BaAl_2_O_4_ crystallizes in a staffed tridymite-type crystal structure^12^,^13^,^14^,^15^ that comprises a three-dimensional network of corner-sharing AlO_4_ tetrahedra with six-membered cavities of the tetrahedral network occupied by alkaline earth ions. In the electron diffraction patterns of its high-temperature phase, characteristic honeycomb-like diffuse scatterings (honeycomb pattern) have been observed^16^. Because the scattering intensities are strongly dependent on the temperature, the characteristic honeycomb pattern may stem from an intrinsic structural fluctuation associated with a soft mode. A recent study of the structural phase transition using synchrotron X-ray diffraction revealed that this system possesses two types of soft modes both of which give rise to strong diffuse scattering intensities which sharply increase towards the structural phase transition temperature (*T*_C_), indicating that both modes condensed simultaneously at *T*_C_^17^. In addition, this compound exhibits an improper ferroelectricity that is accompanied by the structural phase transition at approximately 400 K^12^,^13^,^18^. This transition temperature has been reported to decrease rapidly via the *AE*-site disorder, such as the partial substitution of Sr for Ba^19^. Motivated by the analogy of the fluctuation in the ordered state of spins, charges, or orbitals, we investigated the structural fluctuation in the *AE*-site disordered BaAl_2_O_4_.

In the present study, both the temperature variation of the diffuse scatterings and the structural phase transition temperature for Ba_1−*x*_Sr_*x*_Al_2_O_4_ are systematically studied by transmission electron microscopy (TEM). We report an unusual state lying below 200 K for Ba_1−*x*_Sr_*x*_Al_2_O_4_ with *x* ≥ 0.1 in which the dynamic structural fluctuation develops as the temperature decreases.

## Results and Discussion

**Figure 1:**
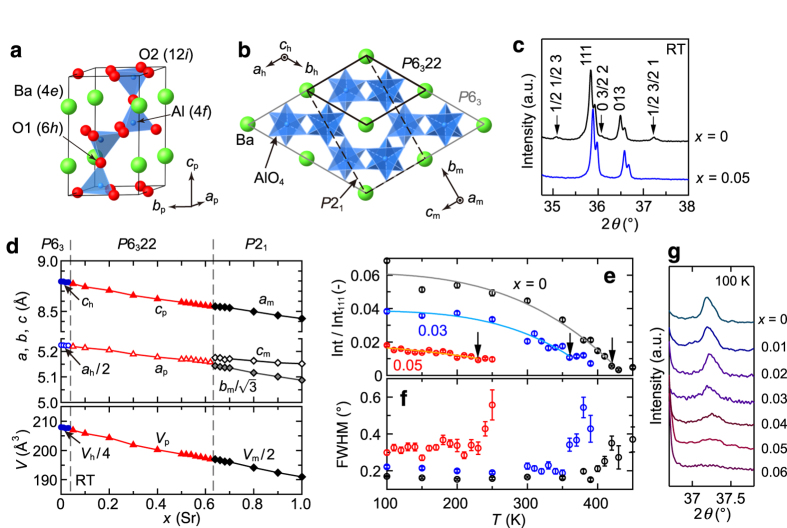
Structural change in Ba_1−*x*_Sr_*x*_Al_2_O_4_. (**a**) Hexagonal *P*6_3_22 parent crystal structure of BaAl_2_O_4_. (**b**) Various cell settings for BaAl_2_O_4_ and SrAl_2_O_4_. The thick solid line and grey line represent the unit cell of the *P*6_3_22 parent structure and the *P*6_3_ low temperature phase of BaAl_2_O_4_, respectively. The broken line shows the *P*2_1_ low temperature phase of SrAl_2_O_4_, which has the same crystal structure as BaAl_2_O_4_ at high temperature. (**c**) Powder XRD profiles in the range of 2*θ* = 35–38° for Ba_1−*x*_Sr_*x*_Al_2_O_4_ (*x* = 0 and 0.05) polycrystalline samples at room temperature. The superlattice reflections are observed in the *x* = 0 sample, as indicated by the arrows. (**d**) Variation of the lattice parameters at room temperature plotted against the nominal Sr-substitution level *x*. The circle, triangle, and diamond symbols represent the lattice parameters of the *P*6_3_, *P*6_3_22, and *P*2_1_ crystal structures, respectively. (**e**) Temperature dependence of the intensity of the 1/2 3/2 1 superlattice reflection normalized by the intensity of the 111 fundamental reflection. Solid lines are fitted curves to show the trend. The error bars are the standard deviations. The arrows indicate the *T*_C_ values. (**f**) FWHM as a function of temperature. (**g**) Powder XRD profiles near the 1/2 3/2 1 superlattice reflection of Ba_1−*x*_Sr_*x*_Al_2_O_4_ with *x* = 0–0.06 obtained at 100 K.

The high-temperature phase of *AE*Al_2_O_4_ (*AE* = Sr or Ba) crystallizes into a hexagonal structure with the space group *P*6_3_22, as shown in Fig. 1a. The low-temperature ferroelectric phase of BaAl_2_O_4_ below *T*_C_ has been identified as a hexagonal *P*6_3_ superstructure with *a*_h_ = 2*a*_p_, *b*_h_ = 2*b*_p_ and *c*_h_* = c*_p_, where the subscripts h and p denote the hexagonal low-temperature structure and the high-temperature parent structure, respectively. SrAl_2_O_4_ crystallizes into the *P*2_1_ monoclinic structure below 950 K^15^. The cell settings of *P*6_3_22, *P*6_3_, and *P*2_1_ are shown in Fig. 1b. The subscript m denotes the *P*2_1_ monoclinic structure. Figure 1c shows the powder X-ray diffraction (XRD) profiles of Ba_1−*x*_Sr_*x*_Al_2_O_4_ (*x* = 0 and 0.05) in the range of 2*θ* = 35–38° at room temperature. The superlattice reflections of *P*6_3_ low-temperature phase, which are marked by arrows, are clearly observed for *x* = 0, whereas they cannot be observed for *x* = 0.05. This indicates that *x* = 0 sample crystallizes in the *P*6_3_ crystal structure at room temperature while *x* = 0.05 sample has the *P*6_3_22 symmetry. High-resolution TEM (HRTEM) experiments for *x* = 0 sample also revealed that the fine antiphase domains with *P*6_3_ symmetry cover the entire area of the crystal, as shown in Supplementary Fig. S1 online. The samples with 0.05 ≤ *x* ≤ 0.62 crystallize in the *P*6_3_22 parent structure at room temperature. Figure 1d shows the lattice parameters at room temperature plotted against the nominal Sr content *x*. A linear decrease in these parameters as *x* increases indicates a systematic substitution of Sr with a smaller ionic radius than Ba. The *P*2_1_ monoclinic structure appears above *x* = 0.64. Thus, we focus on the structural phase transition from *P*6_3_22 to *P*6_3_ for samples with *x* < 0.6.

Figure 1e,f represent the 1/2 3/2 1 superlattice intensities and the full width at half maximums (FWHMs) for *x* = 0, 0.03, and 0.05 samples, respectively, as a function of temperature. The data were obtained during heating. The superlattice intensities are normalized using the intensities of the 111 fundamental reflections (Int_111_). For *x* = 0, the structural phase transition occurs at 420 K, with an abrupt decrease in the superlattice intensity and simultaneous increase in the FWHM. The transition temperature decreases as *x* increases, which is consistent with a previous report^19^. Figure 1g shows the 1/2 3/2 1 superlattice intensities for *x* = 0–0.06 at 100 K. The superlattice intensity decreases and the FWHM increases as *x* increases, as shown in Fig. 1g. This result indicates that the correlation length of the superstructure decreases with increasing the Sr-concentration *x*. For *x* = 0.06, weak intensities of 1/2 3/2 1 resulted in broad peaks even though they were largely independent of temperature. Discontinuities in the lattice parameters could not be detected at the *T*_C_ for each composition.

**Figure 2:**
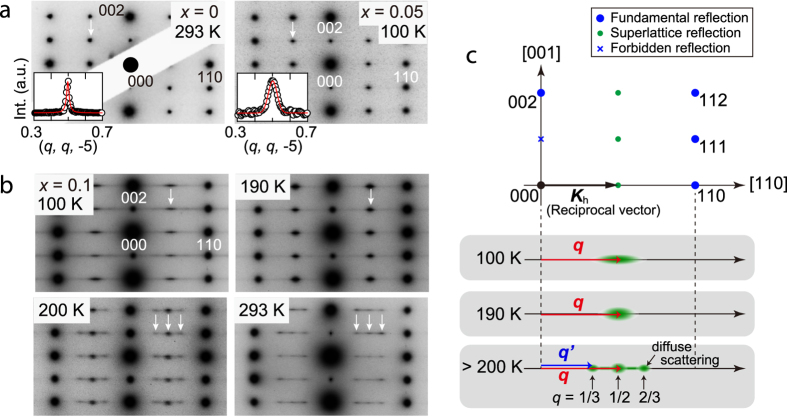
Diffuse scatterings of Ba_1−*x*_Sr_*x*_Al_2_O_4_. (**a**) Diffraction patterns for *x* = 0 single crystal at 293 K (left) and *x* = 0.05 polycrystalline sample at 100 K (right) obtained with a [1–10] incidence. Forbidden reflections with 00*l* (*l* = 2*n* + 1) are observed because of multiple reflections. The insets show the line scans of 1/2 1/2 5– superlattice reflections along [110]. (**b**) Temperature variation of the diffraction patterns with a [1–10] incidence for *x* = 0.1. The arrows indicate the diffuse scatterings. (**c**) Schematic drawings of the relation between a reciprocal vector ***K***_h_ and the wave vectors ***q*** ~ (1/2, 1/2, 0) and ***q***’ ~ (1/3, 1/3, 0).

To investigate the structural fluctuation in the Ba_1−*x*_Sr_*x*_Al_2_O_4_, electron diffraction experiments were performed between 100 and 800 K during heating. Figure 2a shows the [1–10]-zone axis electron diffraction patterns obtained for *x* = 0 at 293 K (left panel) and *x* = 0.05 at 100 K (right panel). The superlattice reflections at the *h* + 1/2 *k* + 1/2 *l* reciprocal positions, marked by arrows in Fig. 2a, indicate the *P*6_3_ superstructure. However, the superlattice reflections for *x* = 0.05 are broader than that for *x* = 0; these reflections for *x* = 0.05 are slightly elongated in the [110] direction. The 1/2 1/2 5– superlattice reflections for *x* = 0 and 0.05 are scanned along [110] in the insets. The FWHM for the superlattice reflection of *x* = 0.05 is clearly larger than that of *x* = 0. Surprisingly, the diffuse scattering appears around the superlattice reflections for *x* = 0 at 310 K, as shown in Supplementary Fig. S2 online, which is 110 K lower than the *T*_C_. Above the *T*_C_, two distinct diffuse scatterings near the *h* + 1/2 *k* + 1/2 *l* and *h* + 1/3 *k* + 1/3 *l* reciprocal positions are observed, as well as diffuse streaks in between them (see Supplementary Figs. S2 and S3 online). Recently synchrotron X-ray diffraction experiments and the first principles calculations have revealed that these diffuse scatterings are ascribed to ***q*** ~ (1/2, 1/2, 0) and ***q***’ ~ (1/3, 1/3, 0) soft modes^17^. That is, two distinct soft modes coexist above the *T*_C_ because of structural instabilities. To the best of our knowledge, this coexistence has not been reported in other compounds thus far.

By contrast, Ba_1−*x*_Sr_*x*_Al_2_O_4_ (0.1 ≤ *x* ≤ 0.5) exhibits an unusual state below ~ 200 K, in which a soft mode fluctuates in ***k***-space. In this state, the low-energy soft mode with the symmetrically equivalent three ***q***s, ***q***_1_ ~ (1/2, 1/2, 0), ***q***_2_ ~ (1, −1/2, 0) and ***q***_3_ ~ (−1/2, 1, 0) in the reciprocal space, deviate from the commensurate ***q*** as the temperature decreases. Figure 2b and Supplementary Fig. S4 online shows the temperature variation of the electron diffraction patterns for *x* = 0.1 obtained during heating from 100 K. Above 200 K, diffuse scatterings near the *h* + 1/2 *k* + 1/2 *l* and the *h* + 1/3 *k* + 1/3 *l* reciprocal positions appear. At 190 K, the diffuse scatterings near *h* + 1/2 *k* + 1/2 *l* reciprocal positions still exist, as indicated by an arrow in the figure, while the diffuse scatterings near the *h* + 1/3 *k* + 1/3 *l* reciprocal positions disappear. Furthermore, these diffuse scatterings change the shape as temperature decreases; they are significantly elongated at 100 K. This strongly indicates that the ***q*** ~ (1/2, 1/2, 0) soft mode does not condensate to form the *P*6_3_ superstructure but survives as a fluctuating state. These changes are schematically shown in Fig. 2c. The significant elongation of the diffuse scattering along [110] implies the deviation of ***q*** ~ (1/2, 1/2, 0) wave vector from the commensurate wave vector. It was confirmed that these changes took place reversibly during a thermal cycle. Similar results were obtained for *x* = 0.3, 0.4, and 0.5.

**Figure 3:**
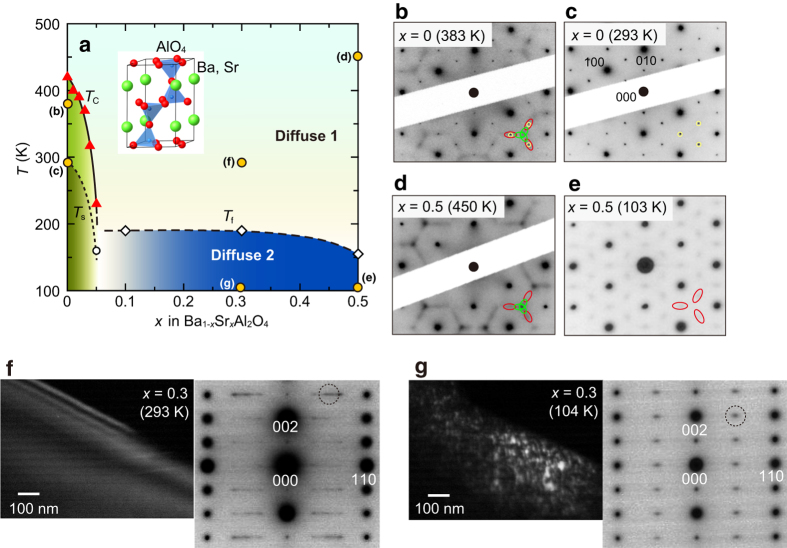
Summary of the results. (**a**) Structural phase diagram for Ba_1−*x*_Sr_*x*_Al_2_O_4_. The closed triangles correspond to the *T*_C_ determined from XRD experiments. *T*_s_ (open circle) for *x* ≤ 0.05 and *T*_f_ (open diamonds) for *x* ≥ 0.1 represent the temperatures below which the diffuse scatterings near the *h* + 1/3 *k* + 1/3 *l* reciprocal positions disappear. (**b**–**e**) Typical electron diffraction patterns for each region. The superlattice reflections are marked by yellow circles in the electron diffraction patterns. In the region above *T*_C_ and *T*_f_, which is denoted as “Diffuse 1”, both the diffuse scatterings near the *h* + 1/2 *k* + 1/2 *l* (red ellipsoids) and *h* + 1/3 *k* + 1/3 *l* (green ellipsoids) reciprocal positions are observed, as well as the diffuse streaks in between them. In the region below *T*_f_, which is denoted as “Diffuse 2”, only the diffuse scatterings near *h* + 1/2 *k* + 1/2 *l* are observed. (**f**,**g**) Dark-field TEM images, including the corresponding electron diffraction patterns at 293 and 104 K, respectively, for *x* = 0.3 using one of the *h* + 1/2 *k* + 1/2 *l* diffuse scattering indicated by the dotted circle.

The structure variation in Ba_1−*x*_Sr_*x*_Al_2_O_4_ is summarized in Fig. 3a together with [001]-zone axis electron diffraction patterns (Fig. 3b–e). The black solid line indicates the *T*_C_ determined from the XRD. The broken line for *x* ≤ 0.05 corresponds to the *T*_s_, below which the diffuse scatterings disappear. Diffuse scatterings near the *h* + 1/2 *k* + 1/2 *l* and *h* + 1/3 *k* + 1/3 *l* positions are observed for temperatures between *T*_C_ and *T*_s_ in addition to the superlattice reflections, as shown in Fig. 3b (see also Supplementary Figs. S2 and S3 online). *T*_f_ denotes the temperature below which the ***q*** vectors of the soft modes deviate from the commensurate value. The coexistence of the two types of diffuse scatterings, which gives rise to the characteristic honeycomb pattern, was observed for a wide temperature region above *T*_C_ and *T*_f_ for all compositions. For *x* = 0.5, this coexistence was observed even at 798 K, as shown in Supplementary Fig. S5 online. This region above *T*_C_ and *T*_f_ is denoted as “Diffuse 1” in Fig. 3a, and the region below *T*_f_ is denoted as “Diffuse 2”.

To gain insight into the structural change at *T*_f_, the microstructure observations were performed on Ba_1−*x*_Sr_*x*_Al_2_O_4_ with *x* = 0.3 using TEM. High-resolution TEM images at 293 K revealed homogeneous lattice fringes with no additional characteristic contrast, such as structural antiphase boundaries (see Supplementary Fig. S6 online). Moreover, fast Fourier transform calculations of the high-resolution TEM images reproduced the experimentally obtained electron diffraction pattern which includes two distinct diffuse scatterings near the *h* + 1/2 *k* + 1/2 *l* and *h* + 1/3 *k* + 1/3 *l* reciprocal positions and diffuse streaks in between them in addition to the fundamental reflections. Figure 3f,g show the dark-field TEM images and the corresponding electron diffraction patterns of the *x* = 0.3 sample obtained at 293 and 104 K, respectively, using one of the *h* + 1/2 *k* + 1/2 *l* diffuse scatterings indicated by dotted circle in Fig. 3f,g under the two-beam condition. There is a homogeneous black region that covers the entire area at 293 K, as shown in Fig. 3f, indicating no superstructure in Diffuse 1 region. Alternating darker and brighter stripes observed in the wedge-shaped region is due to thickness fringes. However, island-like nanoscaled regions with ~20 nm in width are observed as black or white speckles at 104 K, as shown in Fig. 3g. Because the diffuse scatterings near *h* + 1/2 *k* + 1/2 *l* reciprocal positions are strongly dependent on the temperature below ~200 K, it is reasonable to consider that the ***q*** ~ (1/2, 1/2, 0) soft mode still survives in each nanoscaled region.

Because the ***q*** ~ (1/2, 1/2, 0) soft mode does not condensate but instead fluctuate as the temperature decreases, no structural phase transition at lower temperature is expected. Such nanoscaled regions have been observed in several compounds^20^,^21^,^22^,^23^,^24^. For example, in AlV_2−*x*_Cr_*x*_O_4_^20^,^21^, the charge ordered state is suppressed by Cr doping and a spin-glass ground state appears. In TiSe_2_^23^ and Cu_*x*_TiSe_2_^24^, it has been suggested that the onset of superconductivity is associated with the formation of domain walls in the CDW order^25^. Our results strongly suggest the presence of a quantum criticality that the structurally fluctuating state remains intact down to absolute zero temperature without a phase transition. Recently, structural quantum criticality has been discussed for the iron-arsenide superconductor Ba(Fe_1−*x*_Co_*x*_)_2_As_2_, in which the elastic constant softens in the vicinity of superconductivity^26^. Although Ba_1−*x*_Sr_*x*_Al_2_O_4_ is a good insulator, a small amount of nonstoichiometry in Ba and O has been reported in this system^27^. Precise control of this nonstoichiometry is believed to open a path to develop new functional materials that possess a strong coupling of the conduction electrons with the structural fluctuation.

The large structural fluctuation in this compound can be attributed to the intrinsic structural instability caused by the RUMs^8^,^9^,^10^ in the framework compounds with corner sharing polyhedra. These modes are well known in the modifications of SiO_2_, e.g.*,α*-quartz and *β*-tridymite, in which the RUMs act as the soft modes and induce the incommensurate phase transition or the successive phase transition^4^,^5^. In BaGa_2_O_4_ stuffed tridymite-type compound, the intrinsic structural instability is related to the mismatch between the average and interatomic distances^28^. In Ba_1−*x*_Sr_*x*_Al_2_O_4_, a slight change in the structural instability because of atoms occupying the hexagonal channels in a tridymite-type crystal structure would be responsible for the coexistence of the two distinct soft modes in the high-temperature region and for the emergence of the fluctuating low-temperature region dominated by the nanoscaled regions. To clarify the nature of these fluctuating states and the mechanism of the fluctuation in Ba_1−*x*_Sr_*x*_Al_2_O_4_, structural refinements using synchrotron XRD experiments down to the temperature of liquid He are now in progress.

In conclusion, we found that the ordered phase with *P*6_3_ superstructure in BaAl_2_O_4_ is rapidly suppressed by the substitution of Sr for Ba. The unusually fluctuating state with ***q*** ~ (1/2, 1/2, 0) soft mode grows below 200 K in the compositional range between *x* = 0.1 and 0.5. Nanoscaled regions are dominant in this compositional window, indicating that no structural phase transition occurs to the ground state. The intrinsic structural instability in the AlO_4_ tetrahedral network is responsible for this low-temperature fluctuating state.

## Methods

Polycrystalline samples of Ba_1−*x*_Sr_*x*_Al_2_O_4_ (0 ≤ *x* ≤ 1) were synthesized using a conventional solid-state reaction. BaCO_3_ (99.9%), SrCO_3_ (99.9%), and Al_2_O_3_ (99.99%) powder were mixed at a molar ratio of 1:1 and calcined at 1200 °C for 10 h and at 1300 °C for 12 h in air with intermediate grinding. The obtained powder was pressed into a pellet, sintered at 1450 °C for 48 h, and then furnace cooled to room temperature. These pellets were stored in a vacuum. Single crystals of BaAl_2_O_4_ were prepared using a self-flux method in a platinum crucible. A mixture of previously synthesized BaAl_2_O_4_ and BaCO_3_ with a molar ratio of 50:17 was heated at 1470 °C for 6 h and then slowly cooled to 1200 °C at a rate of 2 °C/h. After furnace cooling to room temperature, colourless transparent crystals with a hexagonal shape and with edges approximately 100 μm in length were mechanically separated from the flux. Samples obtained from the flux were stored in a vacuum. Powder XRD was performed using Cu-K*α* radiation in a temperature range between 100 and 500 K. The powder XRD profiles revealed that all of the polycrystalline samples are single-phase samples. Single-crystal XRD experiments also revealed that the crystals have the *P*6_3_ crystal structure at room temperature. *In situ* TEM observations were performed in a temperature range of 100 to 800 K using a double-tilted liquid N_2_ cooling holder and heating holder in the JEM-2010 TEM system (JEOL, Japan). After the desired temperature was reached, all of the diffraction patterns were collected with a time interval of more than 30 min until no changes in the diffraction pattern were observed. All of the indices in the XRD profiles and the electron diffraction patterns are based on the *P*6_3_22 parent phase throughout this paper.

## Additional Information

**How to cite this article**: Ishii, Y. *et al.* A Fluctuating State in the Framework Compounds (Ba,Sr)Al_2_O_4_. *Sci. Rep.* **6**, 19154; doi: 10.1038/srep19154 (2016).

## Supplementary Material

Supplementary Information
