# Supplementary material for: A Fluctuating State in the Framework Compounds (Ba,Sr)Al2O4
Source: Sci Rep. 2016 Jan 13;6:19154. doi: 10.1038/srep19154 (PMC4725368; doi:10.1038/srep19154)
Supplement: Supplementary Information [file srep19154-s1.pdf]

SUPPLEMENTARY INFORMATION TO

A Fluctuating State in the Framework Compounds (Ba,Sr)Al_2O_4

Yui Ishii*, Hirofumi Tsukasaki, Eri Tanaka, and Shigeo Mori

(*Corresponding Author)

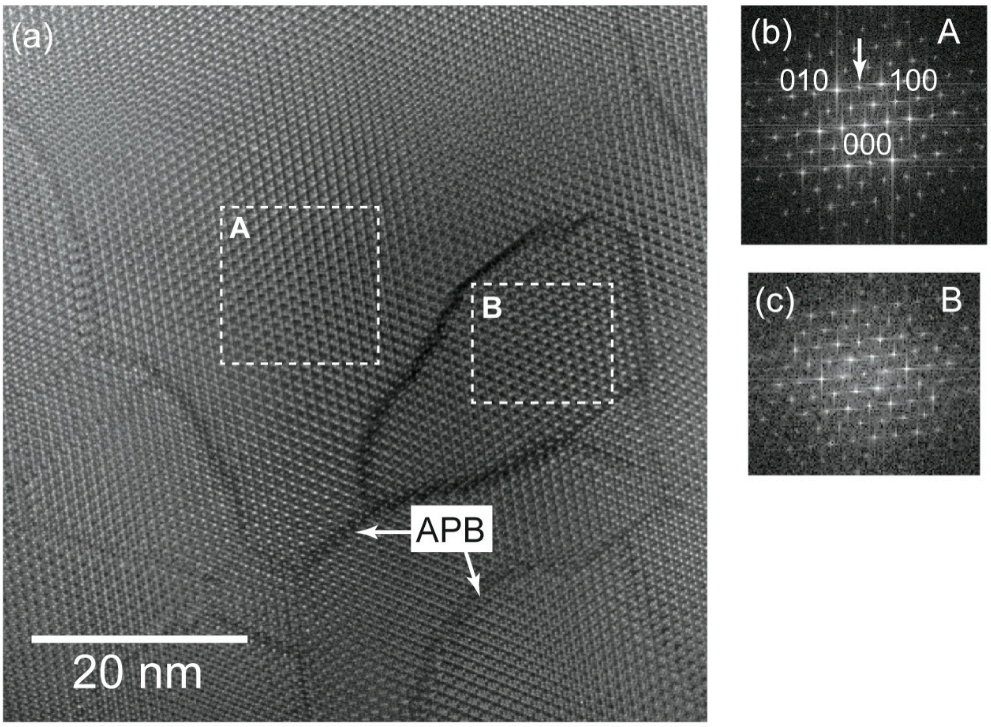

Supplementary Figure S1: (a) High-resolution TEM (HRTEM) image obtained at 298 K in $BaAl_2O_4$ ($x = 0$) with a [001] incidence. In the HRTEM image, meandering black lines can be seen clearly, which are identified as the antiphase boundaries. Fast Fourier transform (FFT) patterns calculated from the regions of A and B are shown in (b) and (c), respectively. The FFT patterns obtained from each domain exhibit the superlattice reflections at the k+1/2 h+1/2 0 positions which indicate the presence of the $2a \times 2b \times c$ superstructure of $P6_3$.

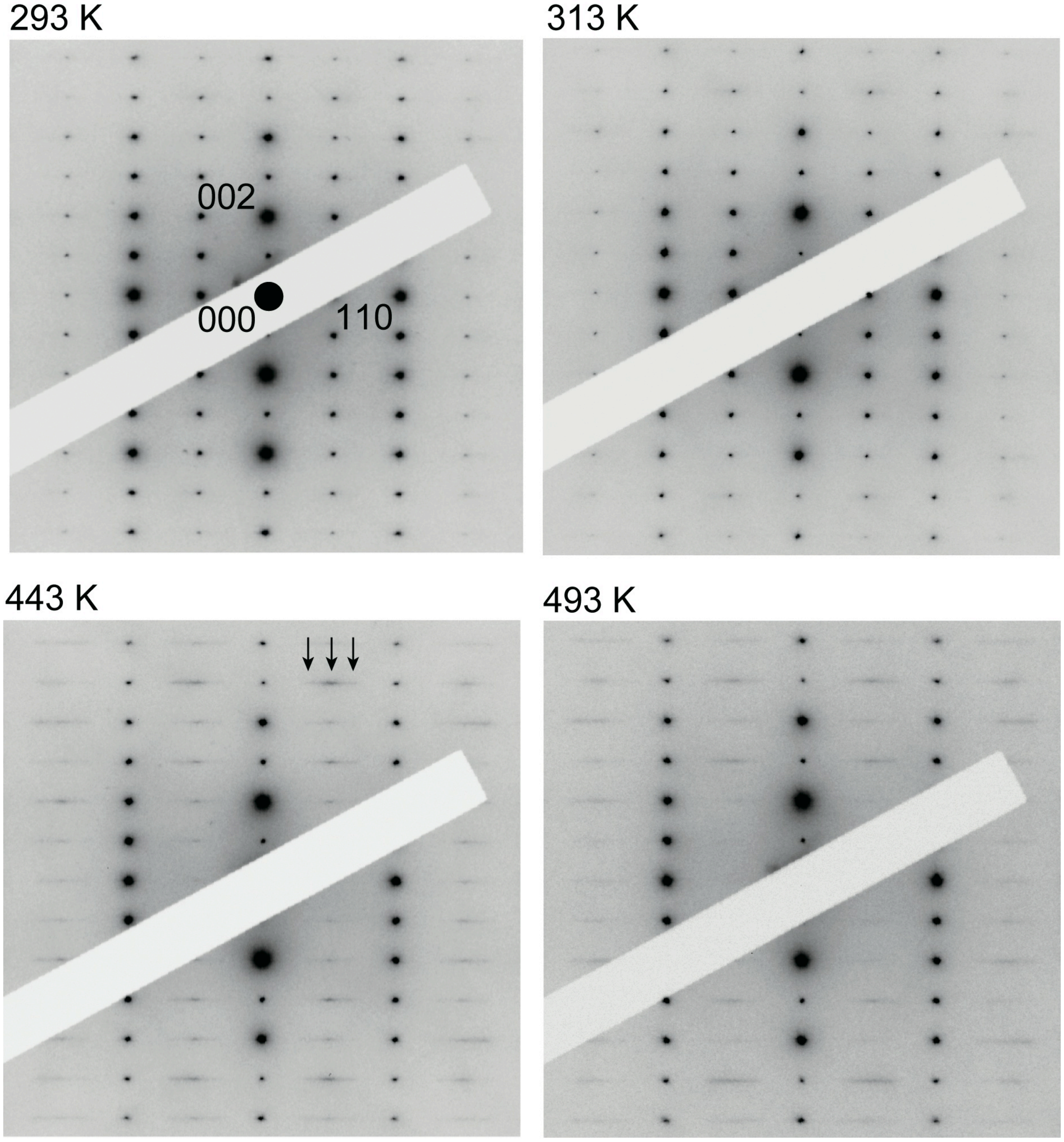

Supplementary Figure S2: Electron diffraction patterns with a [1-10] incidence for $BaAl_2O_4$ (x = 0) single crystal obtained at 293, 313, 443, and 493 K. At 293 K, sharp superlattice reflections are observed as clear spots at (h+1/2, k+1/2, l) reciprocal positions indicating $2a \times 2b \times c$ superstructure. At 313 K, there are weak diffuse scatterings around the each superlattice reflection. At 443 K, there are slightly elongated intensities around (h+1/2, k+1/2, l) and diffuse scatterings around (h+1/3, k+1/3, l), as marked by arrows.

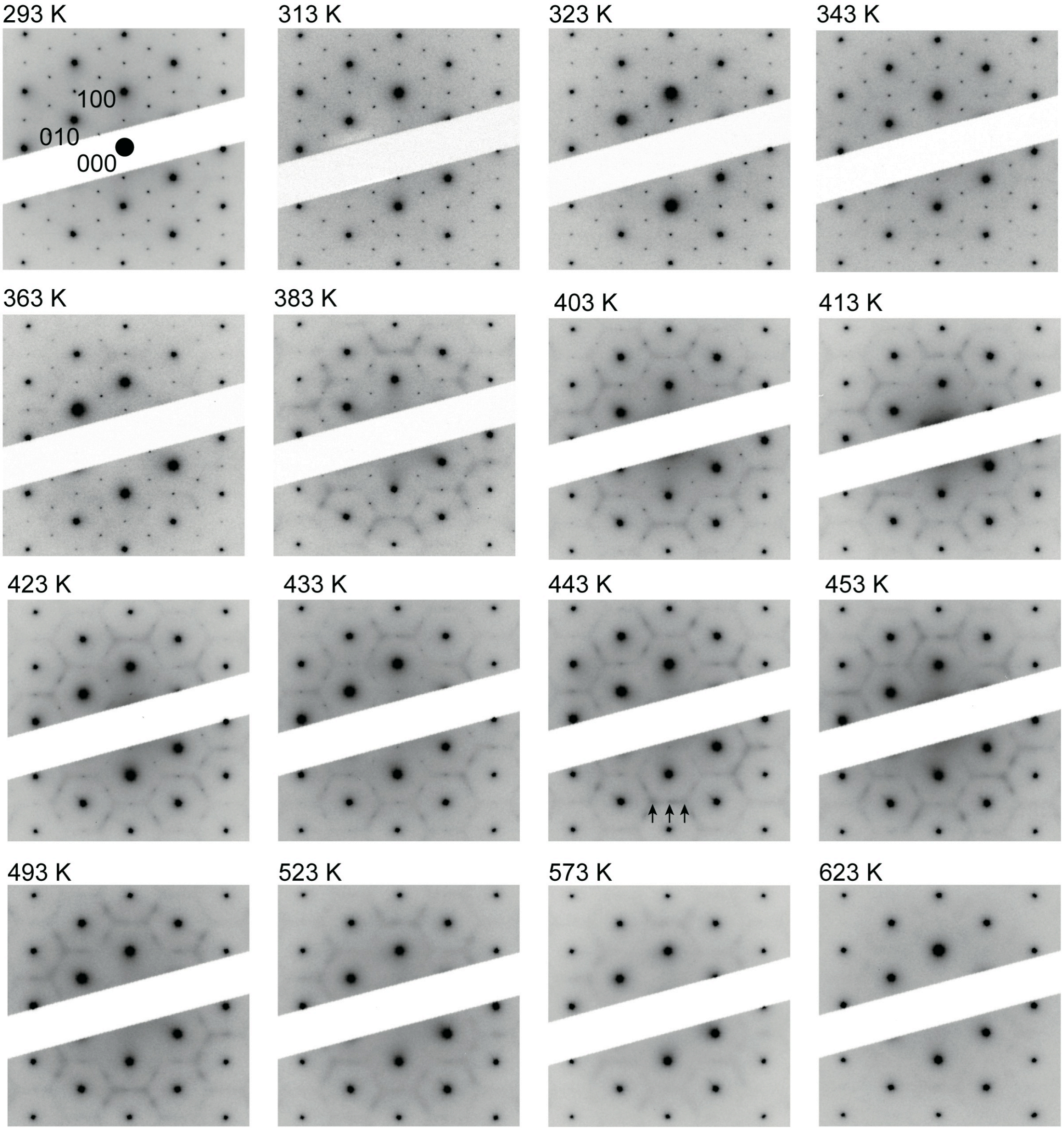

Supplementary Figure S3: Electron diffraction patterns with a [001] incidence for $BaAl_2O_4$ (x = 0) single crystal obtained at 293-623 K. Arrows marks the diffuse scatterings around the (h+1/2, k+1/2, 0) and (h+1/3, k+1/3, 0) reciprocal positions.

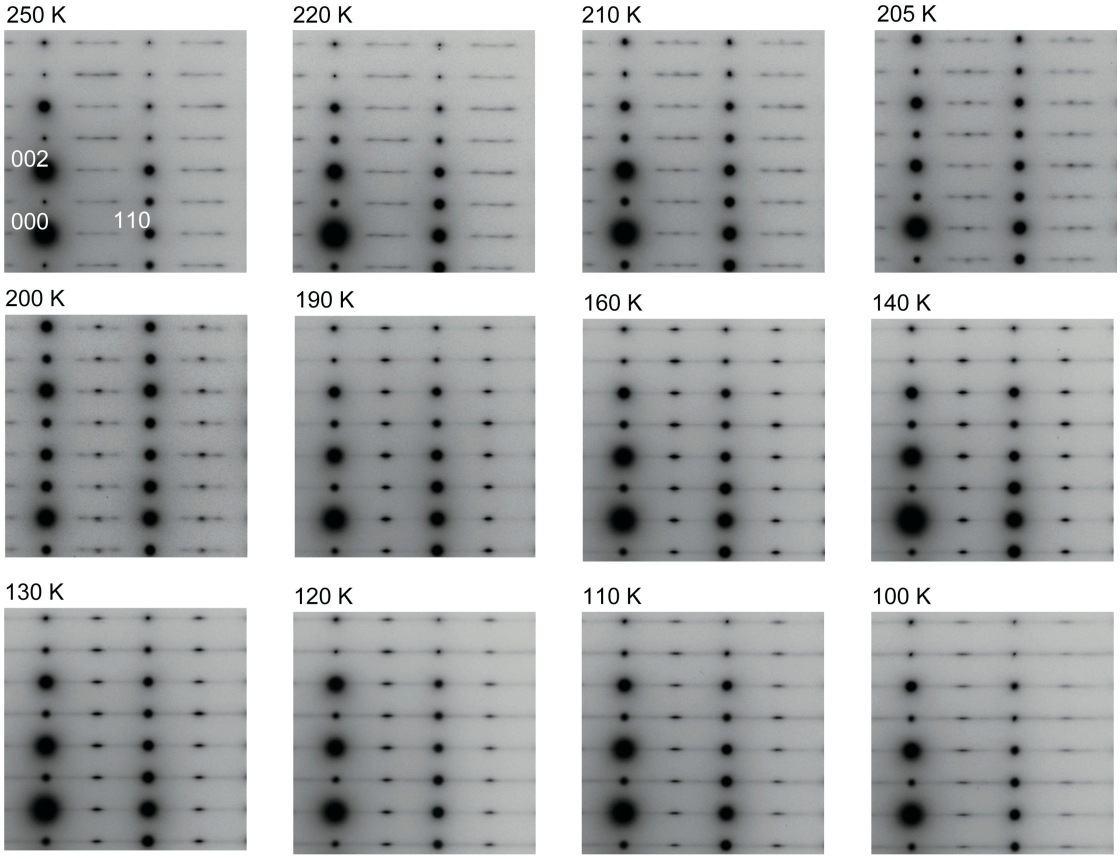

Supplementary Figure S4: Electron diffraction patterns with a [1-10] incidence for $Ba_{1-x}Sr_xAl_2O_4$ (x = 0.1) polycrystalline sample obtained at 250-100 K.

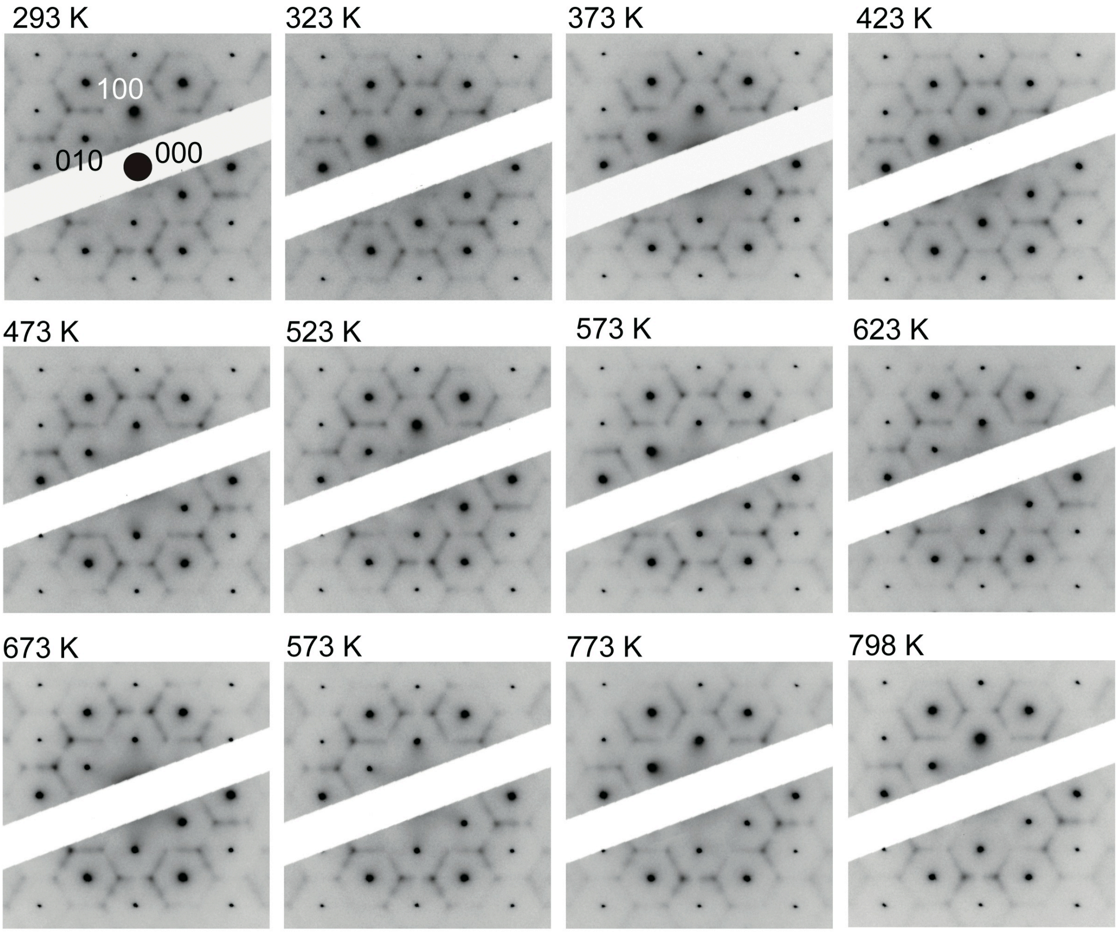

Supplementary Figure S5: Electron diffraction patterns with a [001] incidence for $Ba_{1-x}Sr_xAl_2O_4$ (x = 0.5) polycrystalline sample obtained at 293-798 K.

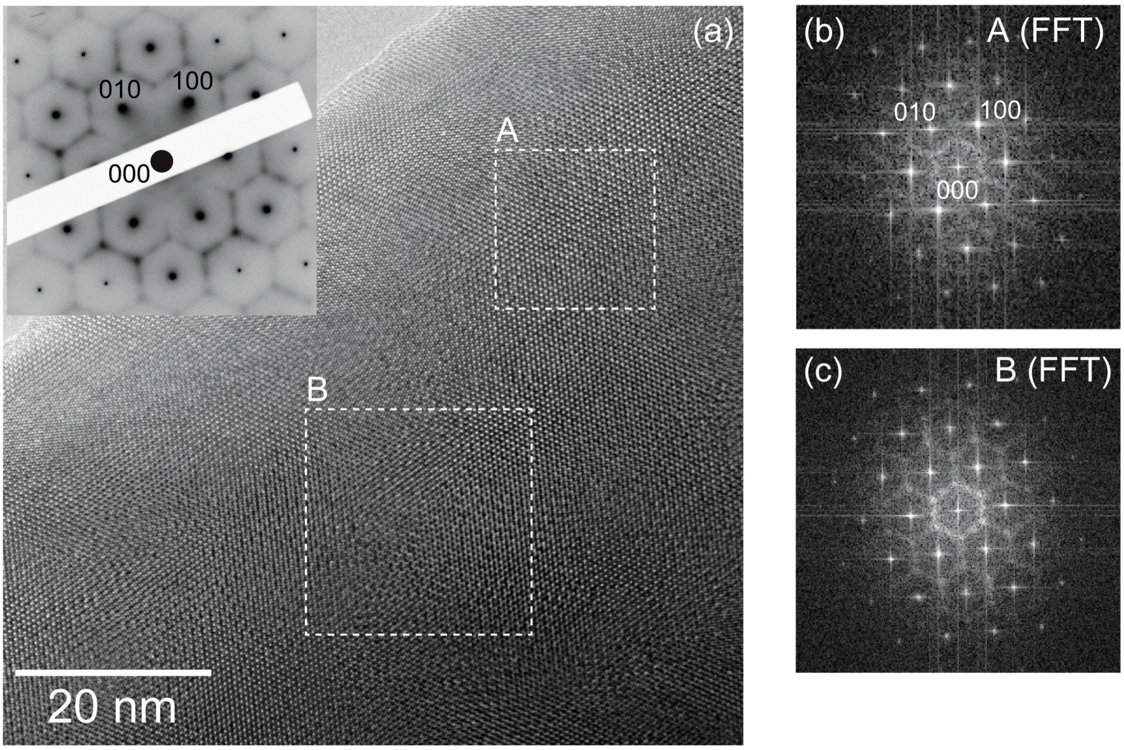

Supplementary Figure S6: (a) HRTEM image obtained at 298 K in $Ba_{1-x}Sr_xAl_2O_4$ (x = 0.3). Inset shows the corresponding electron diffraction pattern obtained experimentally. The HRTEM image reveals homogeneous lattice fringes with no additional characteristic contrast such as antiphase boundaries. FFT patterns calculated from the regions of A and B are shown in (b) and (c), respectively. These FFT patterns reproduce well the experimentally obtained electron diffraction pattern.
